# Case report: Possible role of low-dose PEM for avoiding unneeded procedures associated with false-positive or equivocal breast MRI results

**DOI:** 10.3389/fonc.2024.1405404

**Published:** 2024-07-18

**Authors:** Madeline Rapley, Vivianne Freitas, Irving N. Weinberg, Brandon Baldassi, Harutyun Poladyan, Michael Waterston, Sandeep Ghai, Samira Taeb, Oleksandr Bubon, Anna Marie Mulligan, Alla Reznik

**Affiliations:** ^1^ Department of Physics, Lakehead University, Thunder Bay, ON, Canada; ^2^ Temerty Faculty of Medicine, Joint Department of Medical Imaging, University of Toronto, Toronto, ON, Canada; ^3^ Weinberg Medical Physics, Rockville, MD, United States; ^4^ Radialis Inc., Thunder Bay, ON, Canada; ^5^ Department of Research, Princess Margaret Cancer Centre, University Health Network, Toronto, ON, Canada; ^6^ Laboratory Medicine Program, University Health Network – Toronto General Hospital Site, University of Toronto, Toronto, ON, Canada; ^7^ Thunder Bay Regional Health Sciences Centre, Thunder Bay, ON, Canada

**Keywords:** low-dose positron emission mammography, organ-targeted positron emission tomography, breast MRI, high specificity breast imaging, breast cancer overdiagnosis with MRI

## Abstract

Contrast-enhanced breast magnetic resonance imaging (MRI) is currently recommended as a screening tool for high-risk women and has been advocated for women with radiologically dense breast tissue. While breast MRI is acknowledged for its high sensitivity (with an exception for lower-grade ductal carcinoma *in situ* (DCIS) where emerging techniques like diffusion-weighted imaging offer improvement), its limitations include sensitivity to hormonal changes and a relatively high false-positive rate, potentially leading to overdiagnosis, increased imaging uncertainty, and unnecessary biopsies. These factors can exacerbate patient anxiety and impose additional costs. Molecular imaging with breast-targeted Positron Emission Tomography (PET) has shown the capability to detect malignancy independent of breast density and hormonal changes. Furthermore, breast-targeted PET has shown higher specificity when compared with MRI. However, traditional PET technology is associated with high radiation dose, which can limit its widespread use particularly in repeated studies or for undiagnosed patients. In this case report, we present a clinical application of low-dose breast imaging utilizing a breast-targeted PET camera (Radialis PET imager, Radialis Inc). The case involves a 33-year-old female patient who had multiple enhanced lesions detected on breast MRI after surgical removal of a malignant phyllodes tumor from the right breast. A benign core biopsy was obtained from the largest lesion seen in the left breast. One month after the MRI, 18F-fluorodeoxyglucose (^18^F-FDG) PET imaging session was performed using the Radialis PET Imager. Although the Radialis PET Imager has proven high count sensitivity and the capability to detect breast lesions with low metabolic activity (at a dose similar to mammography), no areas of increased ^18^F-FDG uptake were visualized in this particular case. The patient underwent a right-sided nipple-sparing mastectomy and left-sided lumpectomy, with bilateral reconstruction. The excised left breast tissue was completely benign, as suggested by both core biopsy and the PET results. The case presented highlights a promising clinical application of low-dose breast-targeted PET imaging to mitigate the uncertainty associated with MRI while keeping radiation doses within the safe range typically used in X-ray mammography.

## Introduction

X-ray mammography is the mainstay of breast cancer screening. However, for high-risk women who require screening at a significantly younger age than those at average risk, and for women with radiologically extremely dense breast tissue, the sensitivity of X-ray mammography may be compromised due to the ‘masking effect’ of dense breast tissue. In such cases, breast MRI (1) has been recommended as a supplementary imaging modality (2–5).

Although very sensitive for detecting breast abnormalities before the occurrence of late-stage progression and increasing metastasis-free survival rates (4, 6), contrast-enhanced breast MRI has a number of significant drawbacks (7). These include a high false-positive rate and high sensitivity to hormonal changes, which can result in imaging uncertainty (8–10). These issues contribute to increasing the number of non-cancerous biopsies and may cause overdiagnosis.

Breast-targeted Positron Emission Tomography with 2-[fluorine-18]-fluoro-2-deoxy-D-glucose (^18^F-FDG) as a radiotracer (also known as Positron Emission Mammography (PEM)) is a dedicated molecular (or functional) breast imaging modality (11–14), which detects small cancerous lesions based on their increased glucose metabolism. Moreover, breast-targeted PET is able to detect malignancy independent of breast density (15) and hormonal changes (16), addressing an important limitation of mammography (17, 18).

However, one of the main challenges related to PET imaging is the significant dosage of the injected ^18^F-FDG radiotracer, resulting in higher systemic radiation exposure. As a consequence, the widespread use of PET imaging for breast cancer diagnosis has been limited (16). Here, we present the case of a 33-year-old woman who presented with an increasing solid mass in her right breast. A 3.2 cm lump was excised and found to be a malignant phyllodes tumor with positive margins. Contrast-enhanced breast MRI and low-dose PEM were performed to screen for residual disease. Breast MRI results were positive, showing several bilaterally enhancing masses. The largest lesion in the left breast was biopsied and found to be a benign fibroadenoma. PEM images showed no areas of ^18^F-FDG uptake above the background value in either breast. Based on the MRI results, the patient underwent left-sided lumpectomy despite the negative core biopsy result; according to final pathology report, the large lesion observed in the MRI images was a benign fibroepithelial lesion.

This case demonstrates the possibility of discriminating MRI false-positive lesions through the use of the low-dose breast-targeted Radialis PET Imager, while keeping radiation exposure within the limits used in X-ray mammography. It highlights a promising clinical application of a low-dose PEM device.

## Case description

**Figure 1:**
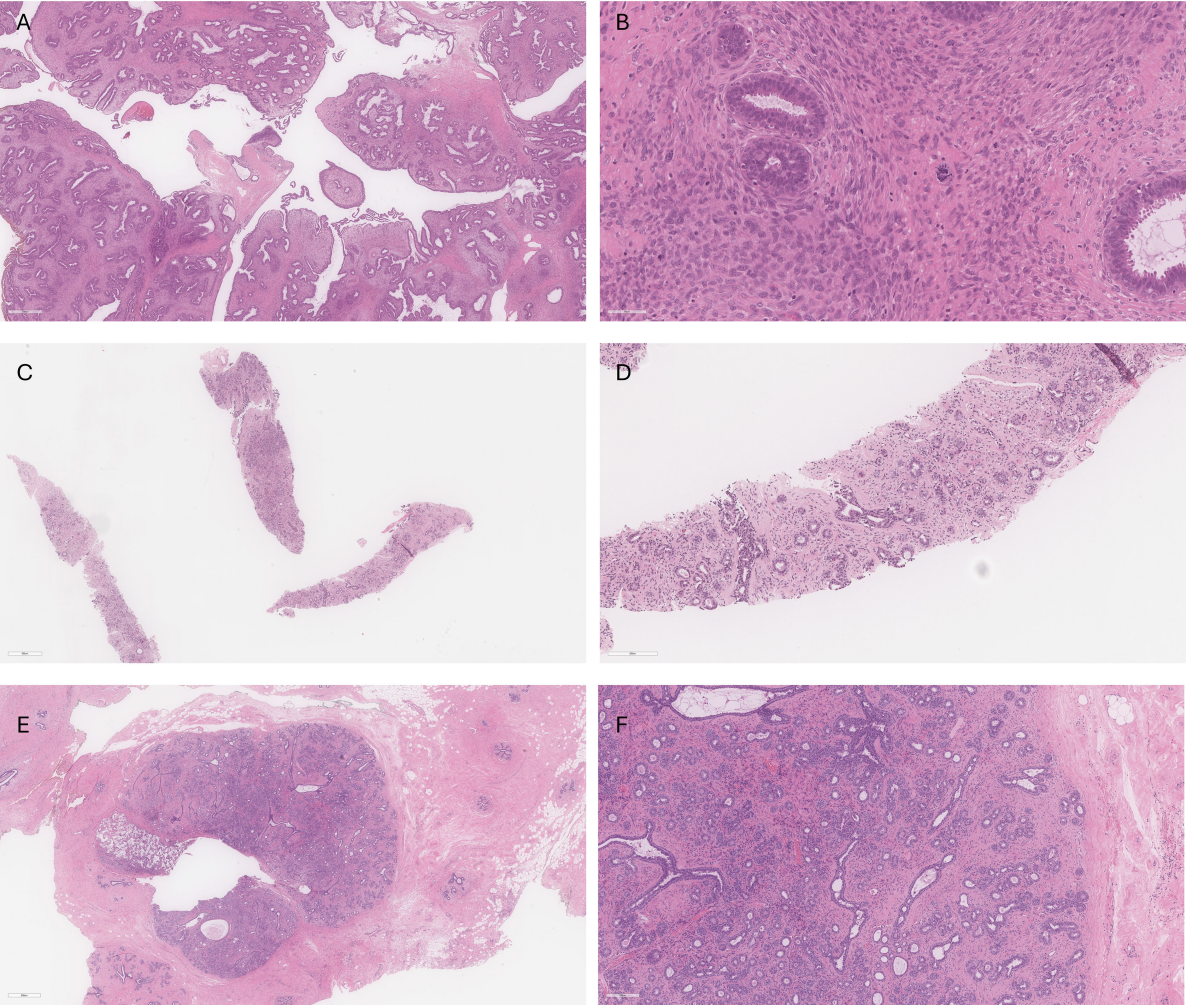
**(A)** Low power view of right breast phyllodes tumor showing cellular leafy fronds composed of benign glands in cellular stroma. **(B)** High power view of right breast phyllodes tumor showing crowding of atypical stromal cells with frequent mitoses (up to 35 mitoses per 10 high power fields) including atypical forms. Grading is challenging in this case given that all features of malignancy are not identified; however, the overall features favored malignant phyllodes tumor in this case. **(C)** Core biopsy of left breast showing a fibroepithelial lesion with mild stromal cellularity. **(D)** Higher power view of left breast core biopsy showing benign glands with small amounts of intervening stroma. The features are compatible with fibroadenoma with some features raising the possibility of tubular adenoma. **(E)** Excision specimen of left breast lesion showing a well-circumscribed fibroepithelial lesion with clip site changes located on the left of the lesion. **(F)** Higher power view of left breast excision specimen demonstrates small round glands with variable amounts of mildly cellular stroma, in keeping with fibroadenoma.

A 33-year-old woman presented with a progressively growing solid mass in her right breast. In September 2020, she underwent a right-sided lumpectomy. The excised lump, identified as a fibroepithelial lesion, along with the fibroepithelial cavity, was sent for pathological analysis. The pathology report revealed a 3.2 cm malignant phyllodes tumor with positive margins (**Figures 1A, B**). A differential diagnosis of a borderline phyllodes tumor was considered due to the lack of marked stromal cytologic atypia, but the presence of several malignant features lead to the diagnosis of malignancy for further management. Phyllodes comprised the entirety of the excised lesion, including multiple of the inked margins. The fibroepithelial lesion contained both uninvolved normal tissue and phyllodes.

**Figure 2:**
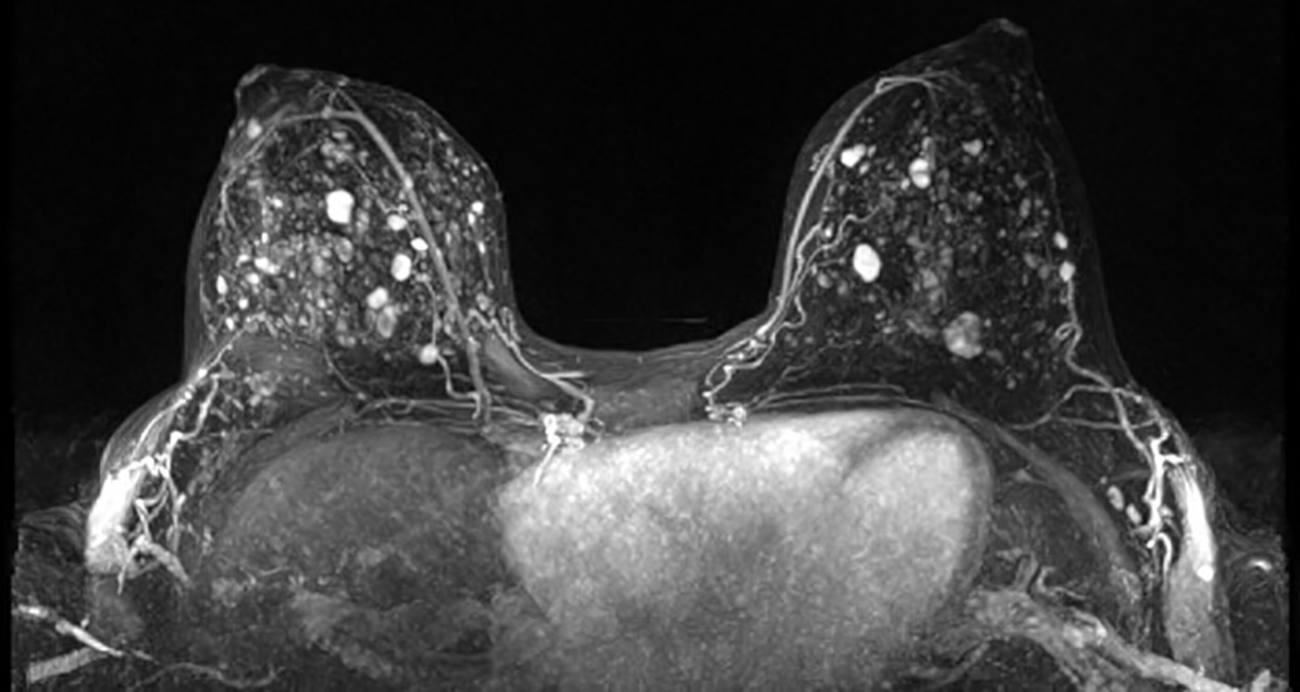
3D-MIP breast MRI showing bilateral enhancing lesions.

52 days after the lumpectomy, the patient underwent an MRI using a dedicated bilateral breast coil. The MRI sequences encompassed pre-contrast axial T1- and T2-weighted images with fat suppression, as well as dynamic contrast-enhanced (DCE) T1-weighted imaging sequences. The DCE sequence included a pre-contrast scan and four post-contrast scans. To minimize the enhancement of benign breast parenchyma, the MRI examination was scheduled during the second week of the menstrual cycle. The images displayed multiple enhanced masses bilaterally, classified as bi-rads 4 (**Figure 2**). A core biopsy was taken from the largest lesion in the left breast visible on MRI (**Figures 1C, D**). The biopsy revealed benign features consistent with a diagnosis of fibroadenoma.

**Figure 3:**
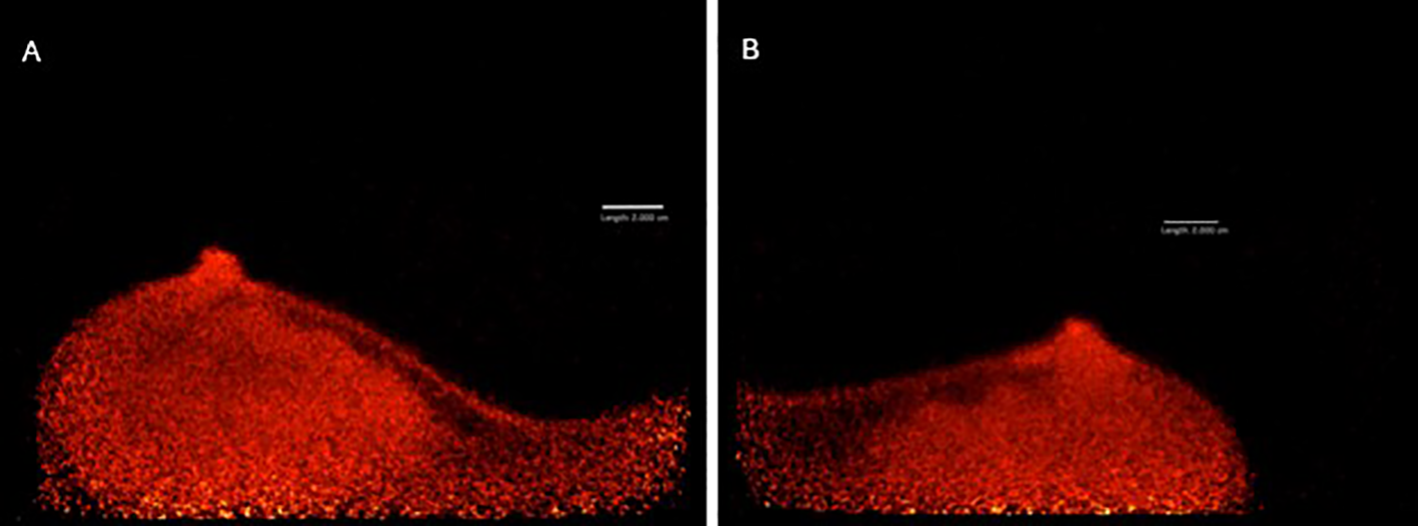
MLO views of left **(A)** and right **(B)** breast acquired with Radialis PET Imager showing no regions of focal ^18^F-FDG uptake.

After obtaining informed consent, the patient agreed to participate in a pilot single-center prospective clinical trial titled “Evaluating Positron Emission Mammography Imaging of Suspicious Breast Abnormalities” (19, 20) (research ethics board no. #18–5029). 79 days after the right-sided surgical excision, the patient was assigned to low-dose (37 MBq) FDG-PET breast imaging. 1 hour after injection, images in the craniocaudal (CC) and mediolateral oblique (MLO) views were acquired using the Radialis PET Imager (**Figure 3**). PET images showed no regions of increased ^18^F-FDG uptake, suggesting no malignancy in either breast.

Despite the core biopsy and PET results, a left-sided lumpectomy was performed due to the suspicious washout pattern observed in the MRI kinetics. This surgery left the patient with breast asymmetry, requiring reconstructive plastic surgery to achieve a satisfactory cosmetic result. Final pathology agreed that the excised lesion was a benign fibroadenoma (**Figures 1E, F**), indicating a false-positive MRI finding and consequent overtreatment. Low-dose imaging with Radialis PET Imager provided accurate results demonstrating its potential clinical applicability to characterize false positive MRI results. With a lower required dose compared to whole body PET, Radialis PET technology may be introduced as a safe and useful tool in this setting. The additional diagnostic information provided by Radialis PET imaging could provide the necessary guidance for conclusive diagnosis and prevent future patients from undergoing needless surgery.

**Figure 4:**
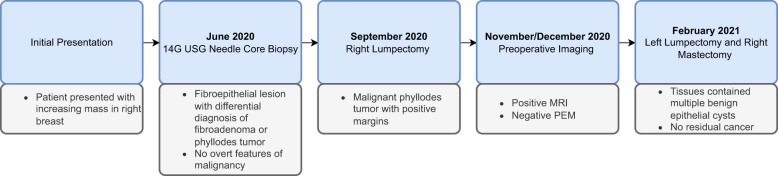
Timeline of the presented case.

This case report follows the CARE (CAse REports) Guidelines. The timeline of the presented case is shown in **Figure 4**.

## Discussion

The Radialis PET Imager has demonstrated the ability to detect breast cancers that were not visible on X-ray mammography, using injected doses that were 90% less than are typically used in whole-body PET (12, 13). At such low injected doses, the radiation exposure to the patient is similar to that for X-ray mammography (21).

In a clinical trial involving 25 patients recently diagnosed with breast cancer (19), the Radialis PET Imager showed a 96% sensitivity, similar to that of MRI, identifying 24 out of 25 invasive cancers. Its false-positive rate was only 16%, significantly lower than the 62% observed for MRI. The dose of the injected ^18^F-FDG varied from 185 MBq (10 participants), to 74 MBq (10 participants), and 37 MBq (5 participants).

The high count sensitivity and signal-to-noise ratio of the Radialis PET Imager allowed a low ^18^F-FDG dose to be administered (37 MBq) (22) addressing concerns related to radiation exposure. The standard dose administered for whole-body PET typically ranges between 370 to 400 MBq, resulting in an effective dose of 6.2–8 mSv (18, 23, 24). To better understand the applicability of radiation medical imaging modalities for screening purposes, it is useful to compare the radiation doses from these examinations with those received from mammography [approximately 0.7 mSv (25), depending on tissue thickness and density, x-ray beam quality and output, and exposure time (26)]. Therefore, not only does 37 MBq of ^18^F-FDG represent a 10-fold reduction compared to the standard whole-body PET dose, but it also remains within the range of the effective dose received during a routine mammographic examination. This opens up the possibility of using low-dose breast-targeted PET for breast cancer screening without exceeding the radiation exposure associated with mammography (22, 27). Given the known issue of high false-positive rates associated with breast MRI, as exemplified by this case, the Radialis PET Imager may be considered as an intermediate step between positive breast MRI and surgery, or possibly as an alternative to MRI as an adjunctive imaging modality to X-ray mammography.

The surgical management of fibroepithelial lesions usually involves a distinct workflow. In the case of phyllodes tumors, surgical excision with a wide margin of at least 1cm is the standard of care (28). Re-excision or mastectomy are recommended for borderline and malignant phyllodes when positive margins are present, as these tumors have a high rate of local recurrence (29, 30). Management of slow growing benign fibroadenomas is typically conservative. MRI is known to have limited capability to distinguish phyllodes tumors from other types of fibroepithelial lesions (28), which in this case lead to surgical excision and subsequent breast reconstruction. By employing a modality with a comparable sensitivity and higher specificity such as low-dose breast-targeted PET, it may be possible to confirm whether surgery is necessary or if a more conservative approach can be taken.

Overall, the presented case underscores the significance of false positive findings that might influence the treatment course, resulting in avoidable surgical interventions. Although the clinical utility of low-dose breast-targeted PET in breast cancer screening and diagnosis should be clarified with prospective clinical trials, the presented case suggests that its inclusion in clinical workflow may be useful when traditional mammography proves insufficient, when there is a high risk of false-positive MRI findings, and when biopsy is challenging or impossible. This will further advance personalized practice in breast cancer diagnosis, potentially providing the additional information required to help prevent life changing overtreatment.

## Conclusion

The importance of early detection in breast cancer cannot be overstated. Mammography has long been recognized as a life-saving tool, and the introduction of breast MRI has further improved our ability to detect tumors in their early stages, particularly in high-risk settings. While the clinical perspective often focuses on the risks of underdiagnosis, patients not only value their lives but also prioritize the preservation of their breasts and the avoidance of unnecessary mastectomies. Striking a balance between effective screening and the potential harm of overdiagnosis is crucial.

By incorporating low-dose breast-targeted PET as a breast cancer diagnostic tool, we can enhance the specificity of tumor detection while reducing the likelihood of unnecessary interventions. This approach aligns with the growing trend of personalized or precision medical imaging, underscoring the significance of saving lives and prioritizing women’s well-being in the pursuit of optimal outcomes in breast cancer screening and diagnosis.

Our case report presents preliminary evidence suggesting that low-dose breast imaging using the Radialis breast-targeted PET camera or similar technology may offer a valuable imaging solution in situations where there is a potential risk of overdiagnosis. Additionally, it could serve as an imaging tool for active surveillance in high-risk patients.

## Data availability statement

The raw data supporting the conclusions of this article will be made available by the authors, without undue reservation.

## Ethics statement

The studies involving humans were approved by University Health Network Research Ethics Board (18-5029, 8 August 2018) Clinical Trial gov Identifier NCT03520218. The studies were conducted in accordance with the local legislation and institutional requirements. The participants provided their written informed consent to participate in this study. Written informed consent was obtained from the individual(s) for the publication of any potentially identifiable images or data included in this article.

## Author contributions

MR: Writing – original draft, Writing – review & editing, Conceptualization, Data curation, Formal analysis, Validation. VF: Conceptualization, Formal analysis, Investigation, Methodology, Validation, Visualization, Writing – original draft, Writing – review & editing. IW: Writing – original draft, Writing – review & editing, Conceptualization, Data curation, Methodology. BB: Formal analysis, Validation, Visualization, Writing – original draft, Writing – review & editing, Software. HP: Formal analysis, Investigation, Writing – original draft, Writing – review & editing, Validation, Visualization. MW: Writing – original draft, Writing – review & editing, Conceptualization, Funding acquisition, Resources. SG: Formal analysis, Investigation, Writing – original draft, Writing – review & editing. ST: Writing – original draft, Writing – review & editing, Formal analysis, Investigation, Project administration. OB: Methodology, Writing – original draft, Writing – review & editing, Supervision, Formal analysis, Investigation. AR: Conceptualization, Funding acquisition, Methodology, Resources, Supervision, Writing – original draft, Writing – review & editing. AM: Writing - original draft, Writing – review & editing, Data curation.

## References

[1] Greenwood HI Wilmes LJ Kelil T Joe BN. Role of breast MRI in the evaluation and detection of DCIS: opportunities and challenges. J Magn Reson Imaging. (2020) 52:697–709. doi: 10.1002/jmri.26985 31746088

[2] Kuhl C Weigel S Schrading S Arand B Bieling H König R. Prospective multicenter cohort study to refine management recommendations for women at elevated familial risk of breast cancer: the EVA trial. J Clin Oncol. (2010) 28:1450–7. doi: 10.1200/JCO.2009.23.0839 20177029

[3] Bakker MF de Lange SV Pijnappel RM Mann RM Peeters PHM Monninkhof EM DENSE Trial Study Group. Supplemental MRI screening for women with extremely dense breast tissue. N Engl J Med. (2019) 381:2091–102. doi: 10.1056/NEJMoa1903986 31774954

[4] Saccarelli CR Bitencourt AGV Morris EA. Breast cancer screening in high-risk women: is MRI alone enough? J Natl Cancer Inst. (2020) 112:121–2. doi: 10.1093/jnci/djz130 PMC7019094 31233125

[5] Berg WA. Current status of supplemental screening in dense breasts. J Clin Oncol Off J Am Soc Clin Oncol. (2016) 34:1840–3. doi: 10.1200/JCO.2015.65.8674 PMC5474360 26962096

[6] Bougias H Stogiannos N. Breast MRI: Where are we currently standing? J Med Imaging Radiat Sci. (2022) 53:203–11. doi: 10.1016/j.jmir.2022.03.072 35469751

[7] Narayanan D Berg WA. Use of breast-specific PET scanners and comparison with MR imaging. Magn Reson Imaging Clin N Am. (2018) 26:265–72. doi: 10.1016/j.mric.2017.12.006 PMC5909988 29622131

[8] Ding W Fan Z Xu Y Wei C Li Z Lin Y. Magnetic resonance imaging in screening women at high risk of breast cancer: A meta-analysis. Med (Baltimore). (2023) 102:e33146. doi: 10.1097/MD.0000000000033146 PMC9997824 36897691

[9] Satoh Y Kawamoto M Kubota K Murakami K Hosono M Senda M. Clinical practice guidelines for high-resolution breast PET, 2019 edition. Ann Nucl Med. (2021) 35:406–14. doi: 10.1007/s12149-021-01582-y PMC7902575 33492646

[10] Rhodes DJ Hruska CB Conners AL Tortorelli CL Maxwell RW Jones KN. Molecular breast imaging at reduced radiation dose for supplemental screening in mammographically dense breasts. AJR Am J Roentgenol. (2015) 204:241–51. doi: 10.2214/AJR.14.13357 PMC4423604 25615744

[11] Weinberg I Beylin D Yarnall S Anashkin E Stepanov P Dolinsky S. (2004). Applications of a PET device with 1.5 mm FWHM intrinsic spatial resolution to breast cancer imaging, in: 2004 2nd IEEE International Symposium on Biomedical Imaging: Nano to Macro (IEEE Cat No 04EX821). Arlington, VA, USA: IEEE. 2:1396–9.

[12] Weinberg IN Beylin D Zavarzin V Yarnall S Stepanov PY Anashkin E. Positron emission mammography: high-resolution biochemical breast imaging. Technol Cancer Res Treat. (2005) 4:55–60. doi: 10.1177/153303460500400108 15649088

[13] Weinberg IN. Applications for positron emission mammography. Phys Med Eur J Med Phys. (2006) 21:132–7. doi: 10.1016/S1120-1797(06)80045-1 17646015

[14] Hathi DK Li W Seo Y Flavell RR Kornak J Franc BL. Evaluation of primary breast cancers using dedicated breast PET and whole-body PET. Sci Rep. (2020) 10:21930. doi: 10.1038/s41598-020-78865-3 33318514 PMC7736887

[15] Ming Y Wu N Qian T Li X Wan DQ Li C. Progress and future trends in PET/CT and PET/MRI molecular imaging approaches for breast cancer. Front Oncol. (2020) 10:1301. doi: 10.3389/fonc.2020.01301 32903496 PMC7435066

[16] Narayanan D Berg WA. Dedicated breast gamma camera imaging and breast PET: current status and future directions. PET Clin. (2018) 13:363–38. doi: 10.1016/j.cpet.2018.02.008 PMC6116731 30100076

[17] Caldarella C Treglia G Giordano A. Diagnostic performance of dedicated positron emission mammography using fluorine-18-fluorodeoxyglucose in women with suspicious breast lesions: a meta-analysis. Clin Breast Cancer. (2014) 14:241–8. doi: 10.1016/j.clbc.2013.12.004 24472718

[18] Chong T. Frost & Sullivan names naviscan a leader in the future of molecular breast imaging. Cancer Wkly. (2009), 860–0.

[19] Freitas V Li X Scaranelo A Au F Kulkarni S Ghai S. Breast cancer detection using a low-dose positron emission digital mammography system. Radiology: Imaging Cancer. (2024) 6:2. doi: 10.1148/rycan.230020 PMC10988332 38334470

[20] Evaluating Positron Emission Mammography Imaging of Suspicious Breast Abnormalities. ClinicalTrials.gov. Available at: https://clinicaltrials.gov/ct2/show/NCT03520218.

[21] Hruska CB O’Connor MK. Curies, and grays, and sieverts, oh my: A guide for discussing radiation dose and risk of molecular breast imaging. J Am Coll Radiol JACR. (2015) 12:1103–5. doi: 10.1016/j.jacr.2015.07.001 PMC4886341 26435124

[22] Stiles J Baldassi B Bubon O Poladyan H Freitas V Scaranelo A. Evaluation of a high-sensitivity organ-targeted PET camera. Sensors. (2022) 22:4678. doi: 10.3390/s22134678 35808181 PMC9269056

[23] Barreto DS Rapelyea JA. Low-dose positron emission mammography: A novel, promising technique for breast cancer detection. Radiology: Imaging Cancer. (2024) 6:2. doi: 10.1148/rycan.240006 PMC10988329 38334472

[24] Kaushik A Jaimini A Tripathi M D’Souza M Sharma R Mondal A. Estimation of radiation dose to patients from 18FDG whole body PET/CT investigations using dynamic PET scan protocol. Indian J Med Res. (2015) 142:721–31. doi: 10.4103/0971-5916.174563 PMC4774069 26831421

[25] Lin EC. Radiation risk from medical imaging. Mayo Clin Proc. (2010) 85:1142–6;quiz 1146. doi: 10.4065/mcp.2010.0260 21123642 PMC2996147

[26] Hendrick RE. Radiation doses and risks in breast screening. J Breast Imaging. (2020) 2:188–200. doi: 10.1093/jbi/wbaa016 38424982

[27] Hruska CB. Let’s get real about molecular breast imaging and radiation risk. Radiol Imaging Cancer. (2019) 1:e190070. doi: 10.1148/rycan.2019190070 33779637 PMC7983654

[28] Zhang M Arjmandi F Porembka J Seiler S Goudreau S Merchant K. Imaging and management of fibroepithelial lesions of the breast: radiologic-pathologic correlation. RadioGraphics. (2023) 43. doi: 10.1148/rg.230051 37856317

[29] Ditsatham C Chongruksut W. Phyllodes tumor of the breast: diagnosis, management and outcome during a 10-year experience. Cancer Manag Res. (2019) 11:7805–11. doi: 10.2147/CMAR.S215039 PMC6707441 31695485

[30] Ranjbar A Zangouri V Shokripour M. Margin status impact on recurrence of phyllodes tumors in high-risk groups: A retrospective observational study. BMC Cancer. (2024) 24:48. doi: 10.1186/s12885-023-11805-2 38195454 PMC10775459

