# Corrigendum: Case report: Possible role of low-dose PEM for avoiding unneeded procedures associated with false-positive or equivocal breast MRI results

**DOI:** 10.3389/fonc.2024.1477862

**Published:** 2024-09-25

**Authors:** Madeline Rapley, Vivianne Freitas, Irving N. Weinberg, Brandon Baldassi, Harutyun Poladyan, Michael Waterston, Sandeep Ghai, Samira Taeb, Oleksandr Bubon, Anna Marie Mulligan, Alla Reznik

**Affiliations:** ^1^ Department of Physics, Lakehead University, Thunder Bay, ON, Canada; ^2^ Temerty Faculty of Medicine, Joint Department of Medical Imaging, University of Toronto, Toronto, ON, Canada; ^3^ Weinberg Medical Physics, Rockville, MD, United States; ^4^ Radialis Inc., Thunder Bay, ON, Canada; ^5^ Department of Research, Princess Margaret Cancer Centre, University Health Network, Toronto, ON, Canada; ^6^ Laboratory Medicine Program, University Health Network – Toronto General Hospital Site, University of Toronto, Toronto, ON, Canada; ^7^ Thunder Bay Regional Health Sciences Centre, Thunder Bay, ON, Canada

**Keywords:** low-dose positron emission mammography, organ-targeted positron emission tomography, breast MRI, high specificity breast imaging, breast cancer overdiagnosis with MRI

In the published article, there was an error in the author list order. When author Anna Marie Mulligan was added, she was erroneously listed as the final author when that position should have been for the primary investigator, Alla Reznik.

The correct author list and affiliations should be:

"Madeline Rapley1 *, Vivianne Freitas 2, Irving N. Weinberg3, Brandon Baldassi 4, Harutyun Poladyan1, Michael Waterston 4, Sandeep Ghai 2, Samira Taeb5, Oleksandr Bubon1,4, Anna Marie Mulligan6 and Alla Reznik1,7."

"1 Department of Physics, Lakehead University, Thunder Bay, ON, Canada, 2Temerty Faculty of Medicine, Joint Department of Medical Imaging, University of Toronto, Toronto, ON, Canada, 3Weinberg Medical Physics, Rockville, MD, United States, 4Radialis Inc., Thunder Bay, ON, Canada, 5Department of Research, Princess Margaret Cancer Centre, University Health Network, Toronto, ON, Canada, 6 Laboratory Medicine Program, University Health Network – Toronto General Hospital Site, University of Toronto, Toronto, ON, Canada, 7Thunder Bay Regional Health Sciences Centre, Thunder Bay, ON, Canada."

The authors apologize for this error and state that this does not change the scientific conclusions of the article in any way. The original article has been updated.

